# LUMEN: A Lightweight UAV Multi-Enhanced Network for PSD-Based RF Fingerprinting on Edge Devices

**DOI:** 10.3390/s26103208

**Published:** 2026-05-19

**Authors:** Min-Joo Yoon, Ki-Woong Park

**Affiliations:** 1 Amgine Inc., 127, Beobwon-ro, Songpa-gu, Seoul 05836, Republic of Korea; mjyoon@amgine.co.kr; 2 SysCore Lab., Sejong University, Seoul 05006, Republic of Korea; 3 Department of Computer and Information Security, Sejong University, Seoul 05006, Republic of Korea

**Keywords:** unmanned aerial vehicle (UAV), radio frequency (RF) fingerprinting, power spectral density (PSD), convolutional neural network (CNN)

## Abstract

Unmanned aerial vehicle (UAV) identification in edge environments requires both high classification accuracy and efficient real-time deployment on lightweight hardware. This study presents LUMEN, a Lightweight UAV Multi-Enhanced Network designed for resource-constrained single-board computer platforms. To enable efficient edge deployment, the proposed method adopts a power spectral density (PSD)-based signal representation together with a lightweight neural network architecture. LUMEN combines multi-channel PSD stacking with multi-scale feature extraction to capture both short-term spectral variations and multi-resolution RF patterns. The proposed pipeline covers UAV RF data collection, including UAV RF data collection, dataset construction, preprocessing, model design, comparative evaluation, and deployment on an RK3582-based edge platform. In the classification experiments, LUMEN achieved the best performance among the four evaluated models, reaching an accuracy of 0.975, compared with 0.775, 0.876, and 0.942 for the baseline models. In the edge deployment test, the model maintained an average inference latency of 1.73 ms and a throughput of 578.95 FPS during a 30-min continuous run, while showing low CPU utilization, low memory usage, and stable thermal behavior. These results demonstrate that LUMEN achieves a practical balance between identification accuracy and runtime efficiency for real-time UAV identification on edge devices.

## 1. Introduction

The rapid proliferation of unmanned aerial vehicles (UAVs) has expanded their applications across agriculture, logistics, and infrastructure monitoring [1,2]. At the same time, the increasing accessibility of low-cost drones has introduced growing security and privacy concerns, including unauthorized surveillance, smuggling, and risks to aviation safety in restricted airspaces [3,4]. These challenges have made reliable UAV identification a critical component of modern counter-UAV systems [2,5].

Traditional detection methods, including radar, thermal imaging, and acoustic sensing, often struggle in urban environments. Small radar cross-sections, environmental noise, and line-of-sight limitations can significantly degrade their performance [6,7]. To address these challenges, Radio Frequency (RF)-based identification has gained attention as a practical alternative. RF fingerprinting exploits hardware-induced imperfections and communication characteristics embedded in UAV control and telemetry signals [8,9,10]. With the adoption of deep learning (DL), recent studies have shown that complex RF patterns can be effectively learned and classified, outperforming conventional rule-based approaches [11,12,13].

Despite these advances, several challenges remain for real-world deployment. First, many existing studies rely on datasets with a limited number of UAV models, which restricts the generalization capability of trained models [14,15]. Second, high-performance DL models often require substantial computational resources, making them difficult to deploy on edge devices with strict power and memory constraints [16,17]. In particular, while Short-Time Fourier Transform (STFT)-based spectrograms are widely used for RF signal representation, their computational overhead can become a bottleneck in real-time systems operating on lightweight hardware [5,18]. These limitations highlight the need for efficient signal representations and lightweight model architectures that can operate reliably on single-board computers (SBCs) [19,20].

To address these issues, this paper proposes LUMEN (Lightweight UAV Multi-Enhanced Network), a lightweight UAV RF fingerprinting approach designed for efficient edge deployment environments. The proposed method adopts a Power Spectral Density (PSD)-based signal representation to reduce preprocessing complexity and latency while preserving discriminative spectral characteristics relevant to UAV identification.

In addition, LUMEN employs a lightweight architecture that integrates multi-channel PSD stacking with multi-scale feature extraction. The multi-channel configuration incorporates adjacent PSD observations to preserve short-term spectral continuity, whereas the multi-scale branches capture spectral patterns across different receptive fields. This design enables the model to simultaneously extract localized spectral variations and broader frequency-domain structures while maintaining lightweight model complexity suitable for edge deployment.

To support this study, we constructed a UAV RF dataset consisting of signals collected from 14 different UAV models, including commercial DJI platforms, hobbyist drones, and custom-built UAVs, providing a diverse evaluation environment. The dataset additionally includes multiple flight states and gain conditions to partially reflect operational signal variability. The proposed approach was further validated through deployment on an RK3582-based edge platform, where real-time performance and system stability were examined under continuous operation conditions.

The main contributions of this study are summarized as follows:

1. UAV RF Dataset Construction: We constructed a dataset comprising RF signals from 14 UAV models, enabling evaluation across diverse hardware characteristics and operating conditions.
2. Lightweight Multi-Enhanced Architecture: We propose LUMEN, which combines multi-scale and multi-channel feature extraction. The model achieves an accuracy of 0.975 while maintaining a compact architecture suitable for edge deployment.
3. Edge Deployment and Real-Time Validation: The proposed system is deployed on an RK3582-based SBC platform. A 30-min continuous test demonstrates stable operation, achieving an average inference latency of 1.73 ms and a throughput of 578.95 FPS, along with stable CPU utilization, memory usage, and thermal behavior.

The remainder of this paper is organized as follows. Section 2 reviews related work on UAV RF fingerprinting and edge deployment. Section 3 describes the dataset construction and preprocessing procedures. Section 4 presents the proposed LUMEN model. Section 5 details the experimental setup. Section 6 presents the experimental results and analysis. Finally, Section 7 concludes the paper.

## 2. Related Work

### 2.1. Signal Representations for RF Fingerprinting

The selection of an appropriate signal representation is a key factor affecting the identification performance of RF-based UAV defense systems [2]. In prior studies, RF signal representations have generally been grouped into three categories: spectral representations, time-frequency representations, and transient-based representations.

Spectral representations emphasize computational efficiency by extracting compact frequency-domain features. Among them, Power Spectral Density (PSD) is widely used because it captures the energy distribution of RF signals in a compact frequency-domain form [7]. Variants such as Welch-based spectral estimation [1] and, in some studies, cepstral features such as MFCC and LFCC [7], have also been explored to improve discriminative capability in multi-class drone detection tasks. In resource-constrained environments, such compact spectral features are often preferred because they offer a practical balance between classification performance and processing overhead [8].

By contrast, time–frequency representations convert 1D RF signals into 2D structures that preserve both spectral and temporal variations. STFT-based spectrograms are among the most widely used representations for this purpose. More recent studies have further explored alternative transformations, including Gramian Angular Fields (GAF) [11] and hybrid representations based on persistence or percentile spectra [21]. Although these 2D representations can provide richer feature patterns and improved robustness in some low-SNR conditions [14], their computational cost can impose significant computational overhead in real-time deployment [2].

Another line of research focuses on transient-based representations, which exploit short-duration hardware-dependent signal characteristics observed during non-steady-state intervals, such as turn-on transients [8,12]. These approaches aim to capture fine-grained hardware fingerprints that may not be clearly visible in steady-state signals [10,22]. Specialized algorithms for precise transient localization are employed to extract fine-grained hardware fingerprints [10]. However, their effectiveness is often sensitive to factors such as sampling rate, synchronization accuracy, and SDR filter design, which can limit their robustness in practical deployment settings [23].

Despite the high accuracy reported in recent deep learning-based UAV identification studies [1,3], achieving both high identification accuracy and real-time throughput on lightweight edge platforms remains challenging. In particular, spectrogram-based approaches often introduce additional preprocessing overhead, which can limit practical edge deployment efficiency.

### 2.2. Lightweight RFFI Models for Edge Deployment

The practical deployment of Radio Frequency Fingerprint Identification (RFFI) systems is often constrained by the computational and memory requirements of deep learning-based models [5,24]. To enable real-time inference on resource-limited edge platforms, recent studies have focused on designing lightweight architectures as well as applying model compression and optimization techniques [16,17,20].

From an architectural perspective, various approaches have been proposed to improve feature extraction efficiency while reducing model complexity. Multi-scale convolutional designs have been widely adopted to capture spectral patterns at different receptive fields with a limited number of parameters [5,24]. In addition, hybrid architectures combining convolutional layers with attention mechanisms have been explored to model both local and global dependencies in RF signals [25]. Temporal convolution-based models, including dilated and causal structures, have also been introduced to efficiently capture long-range dependencies without significantly increasing computational cost [15]. Furthermore, compact recurrent or open-set frameworks have been proposed to address practical deployment scenarios with limited memory budgets [18,26].

In parallel, model optimization techniques play a crucial role in improving edge deployability. Structured pruning and sparse regularization have been shown to significantly reduce model size and computational complexity while maintaining competitive performance [16,17]. Data-level optimizations, such as input truncation and augmentation strategies, have also been adopted to reduce preprocessing overhead and improve generalization under limited data conditions [5,25]. In some studies, lightweight deployment frameworks and model conversion techniques have been utilized to enable execution on embedded platforms [19].

In addition to efficiency, robustness under real-world conditions remains an important consideration. Techniques such as data augmentation, metric learning, and multi-task learning have been employed to enhance generalization performance in noisy and dynamic wireless environments [13,20,27].

Despite these advances, achieving a consistent balance among identification accuracy, lightweight computation, and practical edge deployment remains challenging. Existing approaches often improve only specific aspects, such as feature representation, temporal modeling, or deployment efficiency, rather than jointly considering them in a single model design. Moreover, comprehensive validation under practical RF impairments, such as multipath propagation, Doppler effects, UAV orientation changes, SNR variation, interference, and hardware drift, remains limited in many existing lightweight RFFI studies. Therefore, robustness evaluation under diverse real-world RF channel conditions remains an important open issue for practical UAV RF fingerprinting systems.

### 2.3. Comparative Summary and Research Gap

To clarify the motivation for the proposed architecture, Table 1 summarizes representative RF fingerprinting studies according to their primary design strategies. The comparison focuses on whether each group incorporates PSD-based representations, multi-scale feature extraction, multi-channel modeling, and edge deployment considerations.

As summarized in Table 1, existing studies generally address only part of the overall design space. PSD-based approaches provide computationally efficient spectral representations suitable for lightweight deployment [1,6,7,14], but they often lack sufficient temporal or contextual feature modeling capability. In contrast, spectrogram/image-based deep learning approaches improve representation capability through multi-scale feature extraction [4,5,24,25,26]. However, many of these methods rely on computationally expensive STFT or image-generation pipelines, which increase preprocessing overhead and memory consumption.

Another line of research explores temporal or multi-channel RF modeling using raw IQ sequences [9,15,18,20,26]. These approaches preserve temporal continuity and channel relationships more effectively, but they typically introduce higher computational complexity due to raw IQ processing and sequential modeling structures. Edge-oriented studies further improve deployability through pruning, quantization, and lightweight architectures [16,17,19,24,25], although they mainly focus on compression or acceleration rather than jointly integrating lightweight spectral representation and contextual RF feature extraction.

Based on these observations, an important research gap can be identified: relatively few existing studies jointly investigate the integration of PSD-based lightweight representations, multi-scale feature extraction, multi-channel contextual modeling, and practical edge deployment within a unified architecture. To address this limitation, LUMEN adopts a lightweight PSD-based representation together with multi-channel processing and multi-scale convolutional feature extraction. This design aims to preserve discriminative spectral-temporal information while reducing the computational overhead associated with conventional spectrogram-based RF pipelines.

## 3. Dataset Description and Preprocessing

### 3.1. Dataset Collection Setup

**Figure 1:**
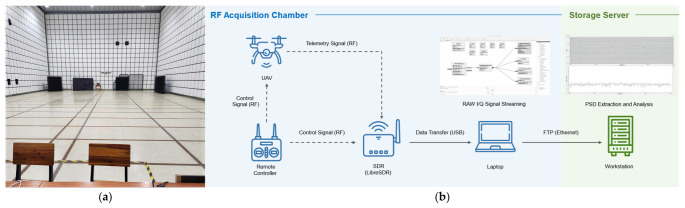
UAV RF data acquisition environment and signal collection pipeline. (**a**) Electromagnetic anechoic chamber used for controlled UAV RF data collection. (**b**) Overall RF signal acquisition and storage workflow, including UAV communication, SDR-based signal capture, and data transfer to the storage server for further processing.

To construct a high-fidelity radio frequency (RF) dataset for UAV identification, we established a controlled data acquisition pipeline. All signal collection was conducted within an electromagnetic anechoic chamber, as shown in Figure 1a, to minimize external interference and ensure controlled signal acquisition conditions. The overall acquisition and storage workflow is illustrated in Figure 1b.

To extract discriminative features from UAV control and telemetry signals, RF data were collected from 14 UAV models (UAV01–UAV14), including commercial DJI platforms, hobbyist drones, and custom-built UAVs, as listed in Table 2. Signal acquisition was performed using a LibreSDR B210 Mini (OpenSourceSDRLab, Guangzhou, China) software-defined radio based on the AD9361 RF transceiver (Analog Devices Inc., Wilmington, MA, USA) in conjunction with GNU Radio Companion version 3.10 (GNU Radio Foundation, Inc., Boston, MA, USA; https://www.gnuradio.org/, accessed on 15 May 2026). The platform provides sufficient RF performance for controlled UAV signal collection at a lower cost compared with conventional USRP-based systems.

The receiver was configured with a bandwidth of 20 MHz and a sampling rate of 20 MS/s. Although many UAV communication systems operate over the wider 2.4 GHz ISM band using frequency hopping spread spectrum (FHSS), this study monitored a 20 MHz instantaneous bandwidth to capture the dominant sub-band where signal activity was most frequently observed during operation. The monitored center frequencies were determined through preliminary spectrum measurements and are summarized in Table 2.

This configuration allowed the system to capture spectral characteristics relevant to UAV identification, including spectral occupancy changes and sideband-related patterns.

To reflect different operating conditions, data were collected under five flight states: arming, takeoff, hovering, landing, and moving. For the arming, takeoff, hovering, and landing states, 12 sessions of 15 s recordings were collected for each condition.

For the moving state, 12 sessions of 40 s recordings were additionally performed to include pitch, roll, and yaw maneuvers, which introduce larger temporal variations in the received RF signals. These moving scenarios were included to partially reflect mobility-related signal variation and dynamic UAV operating conditions during RF acquisition. The overall data collection settings across flight states are summarized in Table 3.

To improve robustness against signal strength variation, data collection was repeated under three indoor gain settings: Indoor01 (40 dB), Indoor02 (70 dB), and Indoor03 (100 dB). During preprocessing, scale factors of 1.0, 0.5, and 0.1 were empirically applied to the corresponding recordings to prevent numerical saturation while preserving relative spectral characteristics across gain conditions.

For each collection condition, RF signals were acquired from both the controller-side control link and the UAV-side telemetry link. As a result, the effective raw recording scale was doubled compared to a single-link acquisition setup. Based on this configuration, the total raw recording time corresponds to 7200 s per UAV and 100,800 s across all 14 UAV models before segmentation and sample selection.

### 3.2. PSD Extraction and Preprocessing

To extract discriminative features from UAV control and telemetry signals, raw in-phase and quadrature (IQ) samples were transformed into a power spectral density (PSD) representation. Compared with time–domain waveforms, PSD representations characterize the distribution of signal power over frequency and are well suited for capturing the spectral characteristics of frequency-hopping spread spectrum (FHSS) and wideband UAV communication signals. In addition, PSD-based representations provide lower preprocessing complexity than spectrogram-based image generation approaches, making them suitable for lightweight edge deployment environments.

In the proposed architecture, the received complex IQ stream is first divided into 100 ms signal segments at a sampling rate of $f_{s}=20$ MHz. The segment duration was selected to capture short-term spectral variation while maintaining stable frequency-domain representations for lightweight processing. Within each segment, PSD features were computed using an FFT length of $N_{FFT}=8192$. This FFT size was empirically selected to provide sufficient frequency resolution while maintaining manageable computational complexity and input dimensionality for edge deployment.

Based on the periodogram estimate, the PSD at the k-th frequency bin is defined as

(1) $$P_{xx}\left(k\right)=\frac{1}{N_{FFT}}{\left|\;\sum_{n=0}^{N_{FFT}-1}s\left[n\right]e^{-j\frac{2\pi kn}{N_{FFT}}}\right|}^{2},$$

where s[n] denotes the discrete-time complex IQ sample and $k\in \{0,1,\ldots ,N_{FFT}-1\}$ is the frequency-bin index. This formulation provides a standardized spectral power estimate for subsequent feature extraction.

To compress the wide dynamic range of spectral power and improve numerical stability during training, the PSD values were further converted into a logarithmic scale:

(2) $$X_{log}(k)=\;10\;{log}_{10}\left(P_{xx}\left(k\right)\;+\varepsilon \right),$$

where ε is a small constant set to $10^{-10}$ to avoid numerical instability when the spectral power approaches zero. Finally, Z-score normalization was applied so that each input feature had zero mean and unit variance.

This preprocessing pipeline emphasizes structural spectral patterns while reducing sensitivity to absolute power fluctuations caused by channel conditions or acquisition environments.

After segmentation, the continuous RF recordings were divided into 100 ms intervals, resulting in approximately 1,008,000 candidate signal segments across all 14 UAV models. Segments containing valid signal activity were screened using an STFT-based signal-presence analysis, and informative samples were retained to ensure balanced representation across flight states for training and evaluation.

As a result, a total of 100,800 signal segments were used for training and evaluation. This selection process reduces redundancy and removes low-information samples while preserving representative spectral characteristics across different operating conditions.

### 3.3. Qualitative Analysis of PSD Patterns

**Figure 2:**
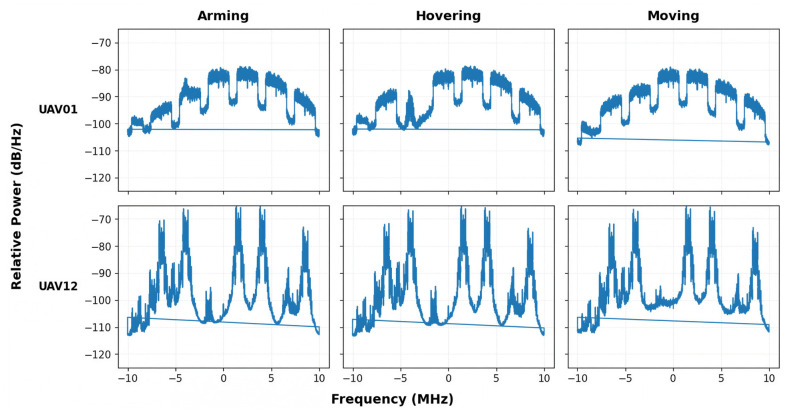
Representative PSD variations across UAV models and flight states. The blue curves represent the PSD magnitude responses over frequency.

To qualitatively examine the discriminative characteristics of the extracted features, PSD representations were compared across UAV models and flight states. Figure 2 shows that the collected signals exhibit clear spectral differences across UAV models, particularly in terms of peak locations, bandwidth occupancy, and sideband-related patterns. These observations indicate that the PSD representation preserves device- and protocol-dependent spectral characteristics useful for UAV identification.

In contrast, the PSD patterns within the same UAV model did not show substantial differences across flight states such as arming, takeoff, hovering, landing, and moving. Although minor state-dependent fluctuations were observed, the overall spectral structure of each UAV model remained relatively consistent across the evaluated flight states. These observations suggest that UAV-specific spectral characteristics constitute the dominant discriminative information in the current dataset, while flight-state variations provide additional intra-class variability during training and evaluation.

The moving condition included pitch, roll, and yaw maneuvers to partially reflect mobility-related signal variation during UAV operation. However, because the data were collected in a controlled environment, these maneuvers should be interpreted as limited mobility variations rather than comprehensive real-world channel dynamics. More extensive experiments under diverse mobility and channel conditions remain necessary for future work.

Overall, the qualitative results in Figure 2 support the use of PSD as an effective input representation for the proposed model. The representation captures both inter-class separability and intra-class variability, which are important for robust UAV classification. The complete set of PSD examples is included in Appendix A.

## 4. Proposed Method

### 4.1. Overall Pipeline

**Figure 3:**
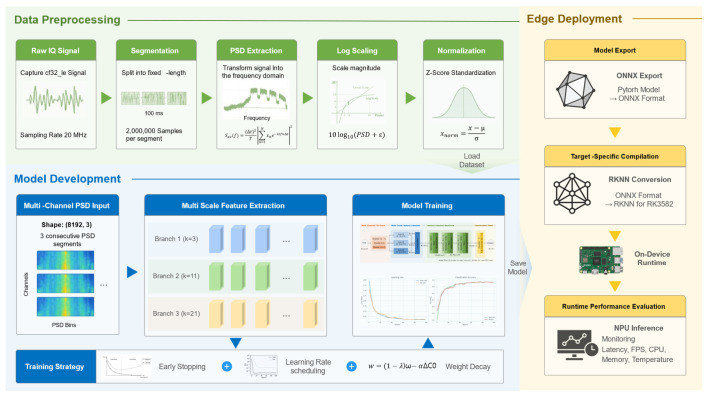
Comprehensive operational pipeline of LUMEN, from signal preprocessing to NPU-optimized edge deployment. Arrows indicate the processing flow and deployment transition between stages.

The proposed pipeline is designed as an end-to-end UAV RF identification framework spanning signal preprocessing, model inference, and real-time edge deployment. As illustrated in Figure 3, the overall pipeline consists of three major stages: data preprocessing, model development, and hardware-aware deployment.

The pipeline begins with the acquisition of raw In-phase and Quadrature (IQ) signals at a sampling rate of 20 MHz. The continuous RF stream is segmented into fixed-length 100 ms intervals, and each segment is transformed into a Power Spectral Density (PSD) representation through the preprocessing pipeline described in Section 3.2. The resulting PSD features are subsequently processed through logarithmic scaling and Z-score normalization to improve numerical stability and training convergence.

To incorporate short-term spectral continuity, three consecutive PSD segments are stacked to form a multi-channel input tensor with a shape of 8192 × 3. The stacked PSD features are then provided as input to the proposed lightweight CNN-based architecture.

The core of the pipeline is LUMEN, which integrates multi-channel spectral input processing and multi-scale feature extraction for UAV RF identification. Multiple parallel convolutional branches are employed to capture spectral characteristics at different receptive fields while maintaining low computational complexity suitable for edge deployment.

During model development, an optimized training strategy was applied, including early stopping, learning rate scheduling, and weight decay regularization. These techniques improved training stability and reduced overfitting during optimization.

For practical deployment, the trained LUMEN model was exported to the ONNX format and subsequently converted into a hardware-specific RKNN format for execution on the Rockchip RK3582 NPU. The deployment pipeline was optimized to maximize hardware parallelism while minimizing CPU overhead.

To evaluate runtime reliability, the deployed system was tested through a 30-min continuous benchmark. During evaluation, inference latency, throughput, and long-term runtime stability were analyzed. These results demonstrate that LUMEN maintains stable high-speed inference and thermal stability, supporting practical UAV identification in resource-constrained edge computing environments.

### 4.2. LUMEN Architecture

The core component of the proposed architecture is LUMEN, a lightweight 1D convolutional neural network designed for UAV signal classification on resource-constrained edge platforms. Unlike conventional 1D CNN architectures that rely on a single PSD segment or a single receptive field, LUMEN combines multi-channel spectral input with multi-scale convolutional processing to capture both local spectral characteristics and short-term spectral continuity.

To motivate the proposed architecture, three baseline models were additionally implemented for comparison:

- A Basic 1D CNN using a single PSD segment and a fixed kernel size;
- A Multi-Scale CNN employing parallel convolutional kernels without temporal-context modeling;
- A Multi-Channel CNN utilizing adjacent PSD segments without multi-scale feature extraction.

As summarized in Table 4, LUMEN integrates both multi-scale and multi-channel strategies by combining the key structural characteristics of the baseline models. Detailed layer configurations of all evaluated architectures are provided in Appendix B.

**Figure 4:**
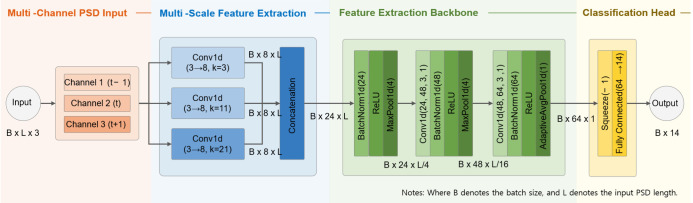
Detailed architecture of LUMEN with multi-channel PSD input and multi-scale 1D convolutional feature extraction.

As illustrated in Figure 4, the input to LUMEN consists of three consecutive PSD segments (t − 1, t, t + 1), forming an input tensor of size (B × 8192 × 3). The three-channel configuration was selected as a compact temporal-context representation by combining the center PSD frame with adjacent observations. This structure enables the model to capture short-term spectral variations while maintaining low computational overhead suitable for lightweight edge deployment. In contrast, using a single PSD segment provides limited temporal context, whereas stacking a larger number of segments substantially increases memory usage and computational complexity.

To extract discriminative spectral features at multiple resolutions, the input is processed through three parallel 1D convolutional branches with kernel sizes of 3, 11, and 21, respectively. The kernel sizes were empirically selected to capture spectral characteristics at different receptive fields while maintaining low model complexity. Smaller kernels primarily focus on localized spectral variations and narrow-band characteristics, whereas larger kernels capture broader spectral structures and wider frequency-domain dependencies. The outputs of the parallel branches are concatenated to generate a fused multi-scale feature representation. 

The fused feature map is subsequently processed through a lightweight backbone composed of max-pooling layers and successive 1D convolutional blocks with Batch Normalization and ReLU activation. This stage progressively reduces the feature dimensionality while preserving discriminative spectral information required for UAV classification.

Finally, adaptive average pooling is applied to generate a compact global feature vector, which is passed to a fully connected layer for 14-class UAV signal classification. Through this architecture, LUMEN effectively captures both multi-resolution spectral characteristics and short-term spectral continuity while remaining suitable for real-time inference on resource-constrained edge devices.

## 5. Experimental Setup

### 5.1. Hardware and Software Environment

Model training was performed on an HP Z440 workstation equipped with an Intel Xeon E5-2690 v4 CPU, an NVIDIA RTX A5000 GPU (24 GB VRAM), 32 GB RAM, and 3 TB HDD storage. The training environment used Rocky Linux 9.6, Python 3.11, PyTorch 2.4.0, and CUDA 12.1. Detailed specifications of the training environment are summarized in Table 5.

For edge deployment and runtime validation, a Radxa ROCK 5C Lite platform based on the Rockchip RK3582 processor was used. The device includes Cortex-A76 and Cortex-A55 CPU cores, 16 GB RAM, and 128 GB microSD storage. The software environment consisted of Ubuntu 24.04.4 LTS, Python 3.10.20, and PyTorch 2.2.0.

ONNX and RKNN-Toolkit2 were used for model conversion, while on-device inference was performed using RKNN-Toolkit-Lite2. UHD 4.6.0.0 was additionally installed to support SDR-based signal acquisition and interfacing. Detailed specifications of the edge deployment environment are summarized in Table 6.

### 5.2. Training Configuration

All models were trained under the same configuration to ensure a fair comparison. The input was constructed from stacked PSD tensors of size 8192 × 3 after logarithmic scaling and Z-score normalization.

For the Basic 1D CNN and Multi-Scale CNN, only the center PSD segment was used as input. In contrast, the Multi-Channel CNN and LUMEN used all three stacked PSD segments to incorporate short-term spectral continuity.

Training was conducted for up to 100 epochs using the Adam optimizer [28] with an initial learning rate of 1 × 10^−3^, a weight decay of 1 × 10^−4^, and a batch size of 64. Cross-entropy loss [29] was adopted for multi-class UAV classification.

To improve training stability, the ReduceLROnPlateau learning-rate scheduler implemented in PyTorch version 2.4.0 [30] was applied with a reduction factor of 0.5 and a patience of 3 epochs. Early stopping with a patience of 10 epochs was additionally used to reduce overfitting during optimization. A summary of the shared training configurations is provided in Table 7.

The statistics used for Z-score normalization were computed from the Indoor01 training dataset and consistently applied to the validation and test datasets.

To prevent data leakage, the dataset was divided at the recording-session level. For each UAV model and flight state, the 12 recording sessions were split into 8 sessions for training, 2 sessions for validation, and 2 sessions for testing. This configuration ensured class balance across all dataset splits.

### 5.3. Evaluation Metrics

The performance of the proposed model was evaluated from three perspectives: classification performance, feature-space quality, and edge deployment efficiency. The evaluation metrics used in this study are summarized in Table 8.

For classification performance, Accuracy, Precision, Recall, and F1-score were used to evaluate overall prediction performance and class-wise behavior. These metrics are derived from the confusion matrix elements: True Positive (*TP*), True Negative (*TN*), False Positive (*FP*), and False Negative (*FN*). The classification metrics are defined as follows:

(3) $$\mathrm{Accuracy}=\frac{TP+TN}{TP+TN+FP+FN},$$

(4) $$\mathrm{Precision}=\frac{TP}{TP+FP},$$

(5) $$\mathrm{Recall}=\frac{TP}{TP+FN},$$

(6) $$F1\text{-}\mathrm{score}=2\times \frac{\mathrm{Precision}\times \mathrm{Recall}}{\mathrm{Precision}+\mathrm{Recall}}.$$

To evaluate the separability and compactness of the learned latent features, three cluster-quality metrics were additionally employed: Silhouette Score, Davies–Bouldin Index (DBI), and Calinski–Harabasz Index (CHI) [31,32,33]. These metrics quantitatively assess how well samples from the same UAV class form compact groups while remaining separated from other classes.

The Silhouette Score evaluates intra-cluster cohesion and inter-cluster separation:

(7) $$\mathrm{Silhouette}\;\mathrm{Score}\;=\;\frac{1}{N}\sum_{i=1}^{N}\frac{b(i)\;-\;a(i)}{max\;\{a\left(i\right),\;b\left(i\right)\}},$$

where *a*(*i*) denotes the average intra-cluster distance and *b*(*i*) represents the average distance to the nearest neighboring cluster.

The Davies–Bouldin Index (DBI) measures the average similarity between clusters:

(8) $$\mathrm{DBI}=\frac{1}{K}\sum_{i=1}^{K}\underset{j\neq i}{\max}\left(\frac{s_{i}+s_{j}}{d\left(C_{i},C_{j}\right)}\right),$$

where *s_i_* and *s_j_* denote intra-cluster dispersion, and *d*(*C_i_*,*C_j_*) represents the distance between cluster centroids.

The Calinski–Harabasz Index (CHI) evaluates the ratio between inter-cluster and intra-cluster dispersion:

(9) $$CHI=\frac{Tr(B_{k})}{Tr(W_{k})}\cdot \frac{N-K}{K-1}$$

where *Tr*(*B_k_*) and *Tr*(*W_k_*) represent between-cluster and within-cluster dispersion, respectively.

Higher Silhouette Score and CHI values indicate better cluster separation and compactness, whereas a lower DBI indicates better clustering quality.

In addition to classification and feature-space evaluation, edge deployment performance was analyzed to assess the practical suitability of the proposed model for real-time UAV identification on resource-constrained hardware. For this purpose, inference latency, throughput, CPU utilization, memory usage, and temperature were measured during on-device execution.

Inference latency represents the processing time for a single input sample, while throughput indicates the number of samples processed per second. CPU utilization and memory usage were monitored to evaluate runtime resource overhead, and temperature was analyzed to assess thermal stability during sustained execution. Together, these metrics provide a complementary evaluation of runtime efficiency and operational robustness in edge environments.

## 6. Results and Analysis

### 6.1. Training Behavior and Convergence

All four models—Basic 1D CNN, Multi-Scale CNN, Multi-Channel CNN, and LUMEN—were trained under identical optimization settings to ensure a fair comparison. Overall, all models exhibited stable learning behavior without significant fluctuations, indicating that the adopted training configuration was appropriate for the UAV RF fingerprinting task.

**Figure 5:**
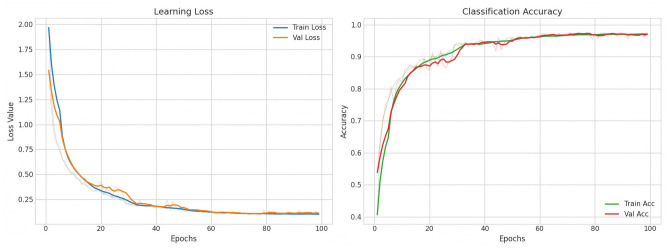
Training and validation results of the LUMEN. The solid curves represent the smoothed training trends, while the faded curves indicate the original unsmoothed values.

Among the evaluated models, LUMEN showed the most stable convergence trend, and its training process is presented in Figure 5. For brevity, the training and validation curves of the three baseline models are provided in Appendix C.

As shown in Figure 5, both the training and validation loss curves decreased steadily, while the corresponding accuracy curves increased and gradually converged. These results indicate that the proposed model effectively learned discriminative spectral features from the PSD-based inputs.

In addition, the gap between training and validation performance remained relatively small throughout training, suggesting limited overfitting. The validation accuracy reached 97.42%, and early stopping was triggered at epoch 99. Overall, the results demonstrate that the proposed model can be trained stably under the adopted optimization settings.

### 6.2. Overall Classification Performance

Table 9 summarizes the classification performance of the four evaluated models. Starting from the Basic 1D CNN baseline, additional architectural components were progressively introduced to analyze the effects of multi-scale feature extraction and multi-channel input processing.

Multi-Scale CNN improved over the baseline model, indicating that multiple receptive fields helped capture diverse spectral characteristics from the PSD representation. Multi-Channel CNN achieved further improvement, suggesting that adjacent stacked PSD segments provided useful short-term spectral continuity information.

Among all evaluated architectures, LUMEN, which combines both multi-scale and multi-channel design strategies, achieved the best overall performance, achieving 0.975 for accuracy, precision, recall, and F1-score. Overall, the results demonstrate a consistent performance improvement as the architecture evolved from the baseline model to the proposed model.

### 6.3. Confusion Matrix and Class-Wise Analysis

**Figure 6:**
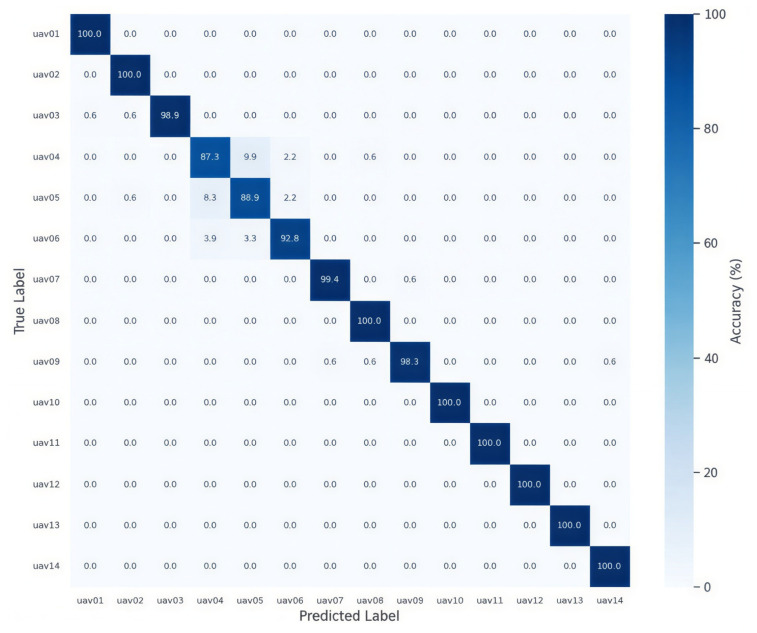
Confusion matrix of the LUMEN for 14-class UAV classification.

Figure 6 presents the confusion matrix of the proposed LUMEN, while the corresponding results for the baseline architectures are provided in Appendix D. Overall, most UAV classes were classified correctly, with only limited off-diagonal errors observed across the confusion matrix.

The most noticeable confusion occurred among UAV04, UAV05, and UAV06. One possible explanation is that UAV01–UAV06 are all DJI platforms and therefore may share similar communication protocols, hardware characteristics, and transmission behaviors. In particular, UAV04, UAV05, and UAV06 were monitored within the same 2.43 GHz frequency range, which may produce similar spectral occupancy and sideband patterns in the PSD representation.

In addition, the dataset includes multiple flight states, including moving conditions with pitch, roll, and yaw maneuvers. These operating conditions can introduce intra-class spectral variation within the same UAV model. Such dataset characteristics may partially explain the localized confusion among spectrally similar DJI UAVs.

Nevertheless, the overall confusion pattern indicates that LUMEN effectively learned discriminative spectral features for most UAV classes and maintained strong class-wise classification performance.

### 6.4. Latent Feature Space Analysis

To evaluate the quality of the learned feature representations, the latent feature spaces of the four evaluated models were analyzed using clustering metrics. Table 10 summarizes the quantitative results, including the Silhouette Score, Davies–Bouldin Index (DBI), and Calinski–Harabasz Index (CHI).

Among the evaluated models, LUMEN achieved the highest Silhouette Score (0.181) and CHI (305.558), along with the lowest DBI (2.242). These results indicate improved cluster compactness and inter-class separability compared with the baseline architectures.

**Figure 7:**
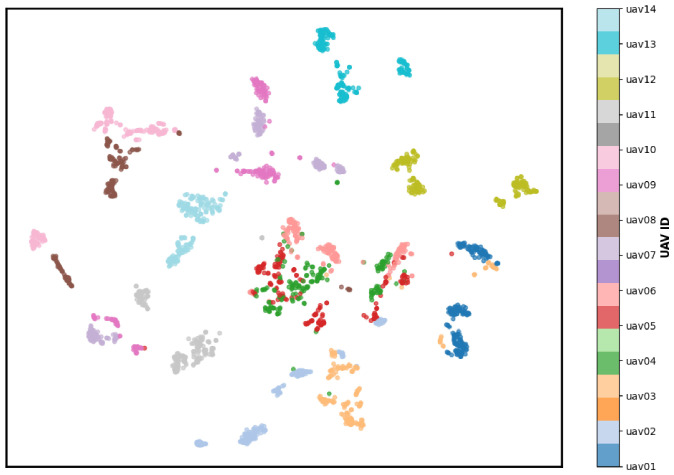
t-SNE visualizations of feature embeddings for the LUMEN.

Figure 7 presents the t-SNE visualization of the feature embeddings generated by LUMEN. Overall, the 14 UAV classes formed relatively well-separated clusters with limited overlap. Although localized mixing was observed among several DJI UAV classes, the overall feature distribution demonstrates meaningful inter-class separation.

The partial overlap among DJI UAV clusters is consistent with the confusion matrix analysis discussed in Section 6.3. Because several DJI UAVs share similar communication protocols and were collected within similar operating frequency ranges, their PSD characteristics may become partially overlapped in the latent feature space. Nevertheless, most UAV classes remained distinguishable, indicating that the proposed model learned robust and discriminative spectral representations.

For comparison, the t-SNE visualizations of the baseline models are provided in Appendix E.

### 6.5. Edge Deployment Evaluation

#### 6.5.1. NPU-Specific Model Optimization and Deployment

To enable real-time inference on the target edge platform, the proposed LUMEN model was deployed on the Rockchip RK3582 NPU using an ONNX-to-RKNN conversion workflow.

The trained model was first exported to the ONNX format [34]. During this process, several implementation details were adjusted to ensure compatibility with the RKNN execution environment. In particular, the deployed model used a fixed input configuration with a stacked PSD tensor of size 1 × 8192 × 3.

The exported ONNX model was subsequently converted using RKNN-Toolkit2 with RK3582 specified as the target platform. After conversion, inference was executed directly on the NPU so that most computational operations could be offloaded from the CPU.

Based on this deployment configuration, runtime behavior was analyzed in terms of inference latency, throughput, hardware resource usage, and long-term operational stability.

#### 6.5.2. Inference Latency and Throughput Analysis

To evaluate whether the deployed model can support real-time operation on the target edge device, a continuous 30 min inference benchmark was conducted on the RK3582 NPU. The benchmark used PSD-based input tensors with a shape of 1 × 8192 × 3, and a total of 993,346 inference iterations were processed during the experiment.

**Figure 8:**
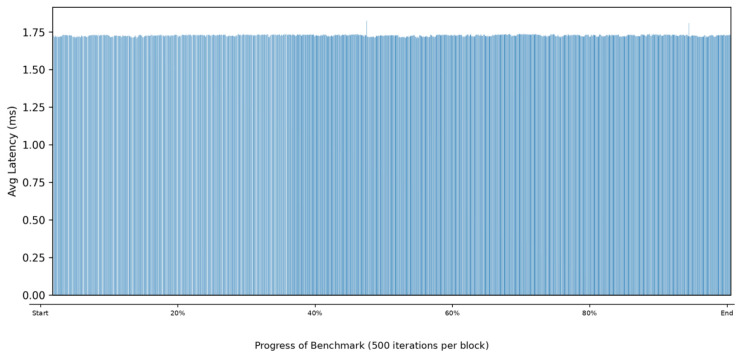
Block-wise average inference latency (500 iterations per block) of the LUMEN model during the 30 min benchmark on the RK3582 NPU.

Figure 8 shows the latency trend throughout the benchmark. Across nearly one million iterations, the inference latency remained consistently around the 1.7 ms range, indicating stable long-duration execution. Although slightly higher latency values were observed during the initial stage, the overall variation remained small and no significant warm-up behavior was observed.

The average inference latency was 1.73 ms, which was identical to the median latency. The 95th and 99th percentile latencies were 1.76 ms and 1.94 ms, respectively, indicating that most inferences were completed within a narrow latency range.

Only a limited number of outliers appeared during the benchmark. Specifically, 23 iterations exceeded 3 ms, and only 2 iterations exceeded 5 ms. Although the maximum observed latency reached 50.77 ms, this occurred only once during the entire experiment and did not affect the overall execution stability.

Table 11 summarizes the inference latency and throughput results obtained during the benchmark.

Based on the measured average latency, the deployed model achieved a throughput of 578.95 FPS. This processing rate is substantially higher than the update frequency typically required for UAV RF identification, providing sufficient computational margin for continuous operation and additional processing tasks on the same platform.

Overall, the latency benchmark demonstrates that the NPU-deployed LUMEN model can sustain stable real-time inference on the Rockchip RK3582 processor (Rockchip Electronics Co., Ltd., Fuzhou, China).

#### 6.5.3. Hardware Resource Efficiency and Thermal Robustness in Edge Environments

**Figure 9:**
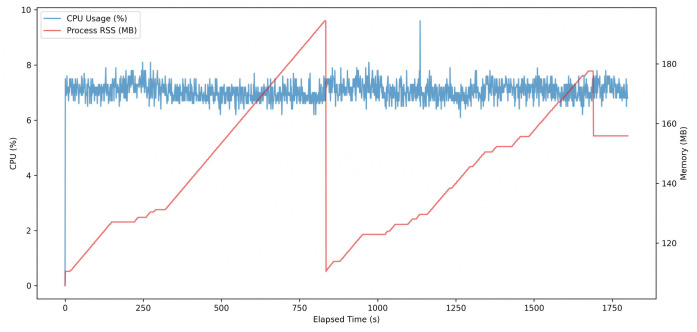
CPU usage and process RSS of the LUMEN model during the 30 min benchmark on the RK3582 NPU.

In addition to latency performance, hardware-level behavior was further analyzed during the same 30 min benchmark. Figure 9 presents the temporal trends of CPU usage and process memory usage, while Table 12 summarizes the corresponding hardware resource and thermal statistics.

As shown in Figure 9, CPU utilization remained low and stable throughout the benchmark. The average CPU usage was 7.05%, indicating that most inference operations were handled by the NPU, while the CPU was mainly responsible for auxiliary runtime tasks.

The process RSS remained within a bounded range of approximately 110–190 MB and did not exhibit a continuously increasing trend over time, indicating stable memory behavior during long-duration inference.

**Figure 10:**
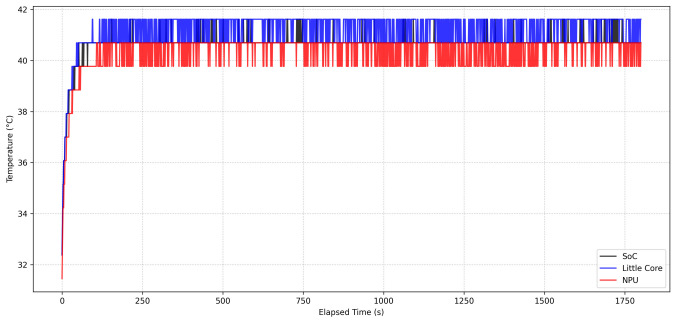
Temperature profiles of the SoC, little CPU core, and NPU during the 30 min benchmark on the RK3582 NPU.

Figure 10 presents the temperature profiles of the SoC, little CPU core, and NPU during execution. After the initial increase from the idle state, all measured temperatures reached stable operating ranges and remained nearly constant throughout the remainder of the benchmark.

As summarized in Table 12, the average SoC and NPU temperatures were 40.65 °C and 40.32 °C, respectively. No noticeable thermal instability or overheating behavior was observed during continuous execution.

Overall, the results demonstrate that the deployed LUMEN model maintained low CPU overhead, bounded memory usage, and stable thermal behavior during continuous real-time inference on the RK3582 platform.

### 6.6. Discussion and Limitations

The experimental results indicate that PSD-based spectral representations can effectively distinguish UAV RF characteristics across multiple UAV categories and flight states. The qualitative PSD analysis and classification results suggest that spectral occupancy patterns, sideband-related structures, and state-dependent spectral variations contribute to inter-class separability within the proposed dataset.

In particular, the inclusion of commercial DJI platforms, hobbyist drones, and custom-built UAVs allowed the proposed approach to learn diverse RF spectral characteristics associated with different communication behaviors and hardware configurations. Furthermore, the moving flight condition, which included pitch, roll, and yaw maneuvers, introduced additional temporal and spectral variability that partially reflects dynamic UAV operating conditions. These dataset attributes contributed to the robustness of the learned spectral representations under varying operating states.

The experimental results also demonstrate that lightweight PSD-based representations combined with multi-scale and multi-channel processing can provide an effective balance between classification performance and real-time edge deployment capability. The proposed model achieved stable runtime behavior on the RK3582 NPU while maintaining relatively low model complexity, demonstrating the practical feasibility of lightweight RF fingerprinting systems for edge computing environments.

Nevertheless, several limitations should be acknowledged.

First, the dataset was collected in an electromagnetic anechoic chamber. Although this environment improves reproducibility and enables clearer observation of UAV-specific spectral characteristics, it does not fully represent real-world outdoor RF environments. In practical deployment scenarios, RF signals may be affected by multipath fading, non-line-of-sight propagation, external interference, and dynamic channel variations, which may influence the robustness and generalization capability of PSD-based UAV identification systems.

Second, several important RF impairments were not systematically modeled or evaluated. Although signal strength variation was partially considered through multiple indoor gain settings and moving flight conditions, the present study did not explicitly incorporate additive noise, co-channel interference, Doppler shifts caused by UAV motion, time-varying fading effects, orientation-dependent channel variation, or long-term hardware characteristic drift. While the proposed dataset partially reflects mobility-related signal variation, the current approach does not explicitly analyze localization-induced variability or complex spatial channel dynamics encountered in real-world deployment conditions.

Finally, the current study provides limited sensitivity analysis for several architectural and preprocessing parameters, including FFT size, segment duration, channel stacking configuration, and kernel size selection in the multi-scale branches. These parameters were empirically selected based on preliminary experiments and computational constraints for lightweight edge deployment. In particular, the three-channel stacking strategy was designed to preserve short-term spectral continuity while maintaining low computational overhead, whereas the multi-scale kernel sizes were selected to capture spectral patterns at different receptive fields. Although the proposed configuration achieved stable performance in the evaluated environment, broader ablation and sensitivity analyses would further improve the interpretability of the architectural design choices and provide deeper insight into the trade-offs between classification accuracy and runtime efficiency.

Future work will focus on extending the dataset to outdoor and interference-rich environments. Additional studies will investigate more realistic RF channel impairments, dynamic spatial conditions, and broader parameter sensitivity analyses to further improve the robustness and practical applicability of lightweight UAV RF fingerprinting systems.

## 7. Conclusions

This paper proposed LUMEN, a lightweight UAV RF fingerprinting approach that integrates PSD-based spectral representation, multi-channel input stacking, and multi-scale 1D convolutional processing. The proposed pipeline was designed to achieve both discriminative UAV identification performance and real-time edge deployment capability.

Experimental results demonstrated that LUMEN achieved an accuracy of 0.975 in the 14-class UAV classification task, outperforming the baseline architectures considered in this study. The confusion matrix and latent feature-space analyses further showed that the proposed model learned discriminative spectral representations across most UAV classes, although partial overlap was observed among several spectrally similar DJI UAVs.

The deployment results on the RK3582 NPU confirmed the practical feasibility of the proposed model for edge environments. During the 30 min benchmark, LUMEN maintained an average inference latency of 1.73 ms and a throughput of 578.95 FPS, while showing stable CPU, memory, and thermal behavior.

Although the current study was conducted under controlled acquisition conditions, the results demonstrate the practical potential of lightweight PSD-based RF fingerprinting for real-time UAV identification. Future work will focus on extending the proposed approach to outdoor and interference-rich environments, incorporating broader RF channel variability, and improving robustness against unseen UAV models.

## Figures and Tables

**Table 1:** Comparison of design strategies in representative RF fingerprinting studies.

Design Strategy	Refs.	PSD-Based	Multi-Scale	Multi-Channel	Edge Eval.	Key Observation
PSD-based representation	[1,6,7,14]	✓	–	–	△	Limited temporal context
Multi-scale feature extraction	[4,5,24,25,26]	–	✓	△	△	Image preprocessing overhead
Multi-channel modeling	[9,15,18,20,26]	–	△	✓	△	Raw-IQ processing overhead
Edge-oriented optimization	[16,17,19,24,25]	△	△	△	✓	Limited spectral- temporal integration
LUMEN	–	✓	✓	✓	✓	Lightweight spectral-temporal integration

Notes: ✓ indicates fully considered, △ indicates partially considered and – indicates not considered.

**Table 2:** Detailed specifications of the target UAV models and monitored center frequencies.

ID	Model Name	Manufacturer (Location)	Group	Monitored Center Freq.
UAV01	Mavic 3 Classic	DJI (Shenzhen, China)	DJI	2.43 GHz
UAV02	Phantom 3 SE	DJI (Shenzhen, China)	DJI	2.43 GHz
UAV03	Air 2S	DJI (Shenzhen, China)	DJI	2.43 GHz
UAV04	Mini 4 Pro	DJI (Shenzhen, China)	DJI	2.43 GHz
UAV05	Neo	DJI (Shenzhen, China)	DJI	2.43 GHz
UAV06	Avata 2	DJI (Shenzhen, China)	DJI	2.43 GHz
UAV07	X5	Syma (Shantou, China)	Hobbyist	2.45 GHz
UAV08	Aerial Drone	Daeho Toys (Seoul, Republic of Korea)	Hobbyist	2.45 GHz
UAV09	HS210	Holy Stone (Shenzhen, China)	Hobbyist	2.45 GHz
UAV10	H235	Shenzhen Yunwei Elec. (Shenzhen, China)	Hobbyist	2.45 GHz
UAV11	HHSSL 4K	HHSSL (Nanjing, China)	Hobbyist	2.475 GHz
UAV12	Bee35	Custom-built (Seoul, Republic of Korea)	Custom	2.45 GHz
UAV13	Pixhawk PRO	Custom-built (Seoul, Republic of Korea)	Custom	2.45 GHz
UAV14	MARK4	Custom-built (Seoul, Republic of Korea)	Custom	2.45 GHz

**Table 3:** Summary of data collection settings and raw recording scale across flight states.

Flight State	Sessions	Duration	Gains	Total Recorded Time
Arming	12	15 s	40/70/100 dB	1080 s
Takeoff	12	15 s	40/70/100 dB	1080 s
Hovering	12	15 s	40/70/100 dB	1080 s
Landing	12	15 s	40/70/100 dB	1080 s
Moving	12	40 s	40/70/100 dB	2880 s
Total (per UAV)				7200 s

**Table 4:** Structural complexity and parameter comparison of the evaluated models.

Model	Multi-Scale	Multi-Channel	Total Params
Basic 1D CNN	–	–	718
Multi-Scale CNN	✓	–	5890
Multi-Channel CNN	–	✓	9070
LUMEN	✓	✓	14,830

Notes: ✓ indicates fully considered and – indicates not considered.

**Table 5:** Specifications of model training environment.

Type	Category	Specification
HW	Computing Platform	HP Z440 Workstation
	CPU	Intel Xeon E5-2690 v4 @ 2.60 GHz
	GPU	NVIDIA RTX A5000 (24 GB VRAM)
	Memory	32 GB RAM
	Storage	3 TB HDD
SW	Operating System	Rocky Linux 9.6
	Programming Environment	Python 3.11
	Deep Learning Framework	PyTorch 2.4.0
	GPU Acceleration	CUDA 12.1

**Table 6:** Specifications of edge deployment environment.

Type	Category	Specification
HW	Computing Platform	Radxa ROCK 5C Lite
	Processor	Rockchip RK3582
	CPU	2 × Cortex-A76 + 4 × Cortex-A55
	Memory	16 GB RAM
	Storage	128 GB SD Card
SW	Operating System	Ubuntu 24.04.4 LTS
	Programming Environment	Python 3.10.20
	Deep Learning Framework	PyTorch 2.2.0
	Model Conversion	ONNX, RKNN-Toolkit2 2.3.2
	Inference Runtime	RKNN-Toolkit-Lite2 2.3.2
	SDR Support Library	UHD 4.6.0.0

**Table 7:** Shared configurations for model training.

Category	Parameter	Specification
Common Setup	Optimizer	Adam (weight decay = 1 × 10^−4^)
	Loss function	Cross-entropy loss
	Learning rate	Initial LR = 1 × 10^−3^
	LR scheduler	ReduceLROnPlateau (factor = 0.5, patience = 3)
	Max epochs	100
	Early stopping	Patience = 10 epochs
	Batch Size	64
Model Config.	Raw Input	PSD tensor (8192 × 1 or 8192 × 3)
	Preprocessing	Log scaling + Z-score normalization
	Normalization	Mean/std computed from Indoor01 Training data
	Output classes	14 UAV classes

**Table 8:** Evaluation metrics for performance assessment.

Category	Metric	Description
Classification	Accuracy	Overall correct predictions
	Precision	Correct positive predictions
	Recall	Correctly identified actual positives
	F1-score	Harmonic mean of precision and recall
Feature Space	Silhouette Score	Intra-class cohesion and inter-class separation
	Davies–Bouldin Index	Average cluster similarity
	Calinski–Harabasz Index	Ratio of between-/within-cluster dispersion
Edge Deployment	Latency	Time required to process one input sample
	Throughput	Number of samples processed per second
	CPU Utilization	Host CPU usage during inference
	Memory Usage	Runtime memory consumption during inference
	Temperature	Thermal stability during sustained operation

**Table 9:** Classification performance of the four evaluated models.

Model	Accuracy	Precision	Recall	F1-Score
Basic 1D CNN	0.775	0.777	0.775	0.767
Multi-Scale CNN	0.876	0.880	0.876	0.877
Multi-Channel CNN	0.942	0.942	0.942	0.942
LUMEN	0.975	0.975	0.975	0.975

**Table 10:** Quantitative analysis of feature space representation for four models.

Model	Silhouette Score ↑	Davies–Bouldin Index ↓	Calinski–Harabasz Index ↑
Basic 1D CNN	0.020	3.680	174.634
Multi-Scale CNN	0.060	3.962	153.590
Multi-Channel CNN	0.123	2.767	254.536
LUMEN	0.181	2.242	305.558

Notes: ↑ indicates higher is better and ↓ indicates lower is better.

**Table 11:** Inference latency and throughput statistics of the LUMEN model on the RK3582 NPU.

Metric	Initial Phase (Avg.)	Overall Average	Maximum Overhead
Inference Latency	1.8 ms	1.73 ms	50.77 ms
Throughput (FPS)	~555 FPS	578.95 FPS	-

**Table 12:** Hardware resource usage and thermal statistics of the LUMEN model on the RK3582 NPU.

Category	Metric	Value
Resource usage	Average CPU usage	7.05%
	Process RSS range	110–190 MB
Thermal	Average SoC temperature	40.65 °C
	Average NPU temperature	40.32 °C

**Figure A1:**
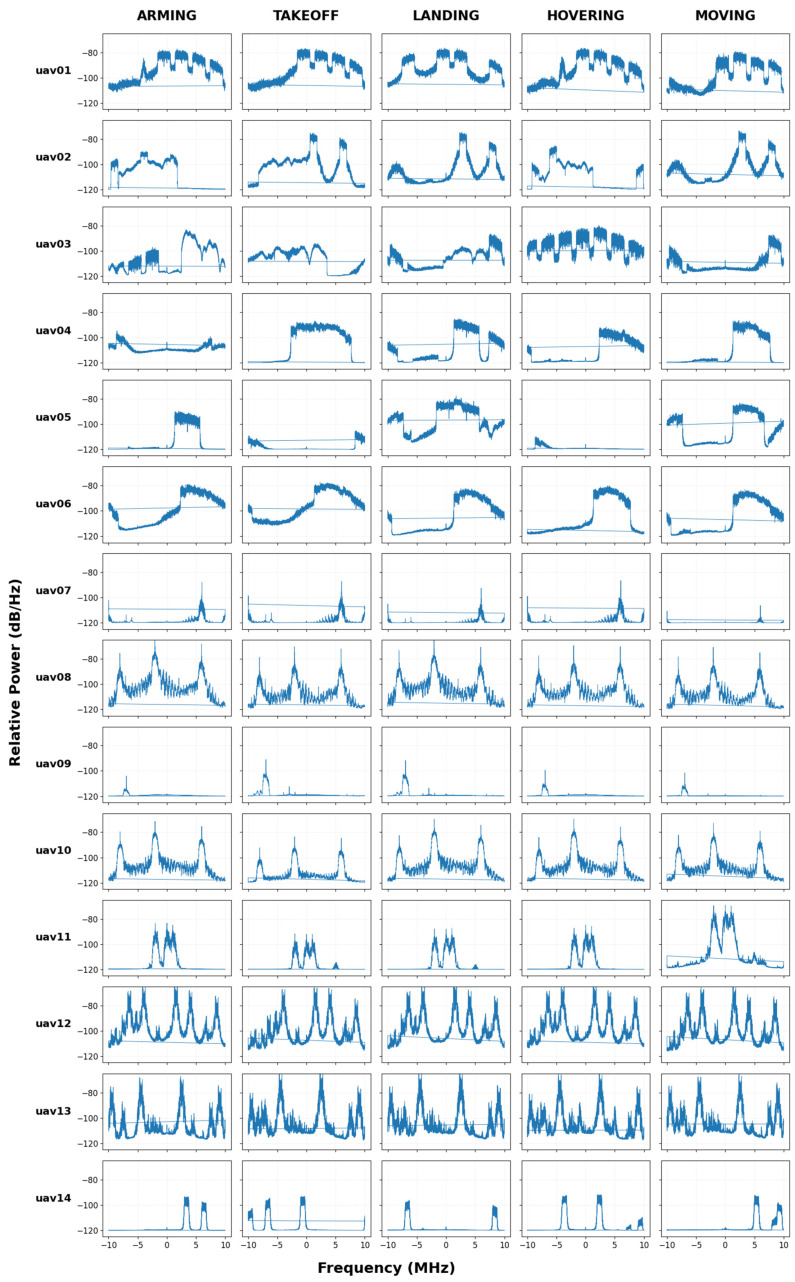
PSD visualizations of 14 UAV models across five flight states. The blue curves represent the PSD magnitude responses over frequency.

**Figure A2:**
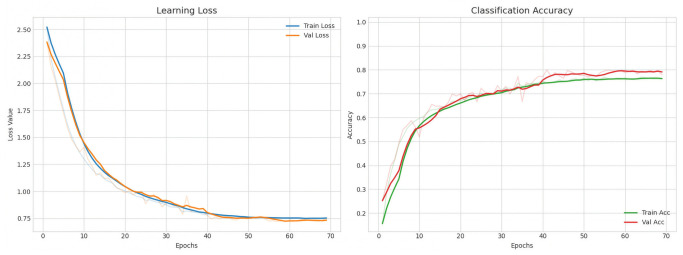
Training and validation results of the Basic 1D CNN. The solid curves represent the smoothed training trends, while the faded curves indicate the original unsmoothed values.

**Figure A3:**
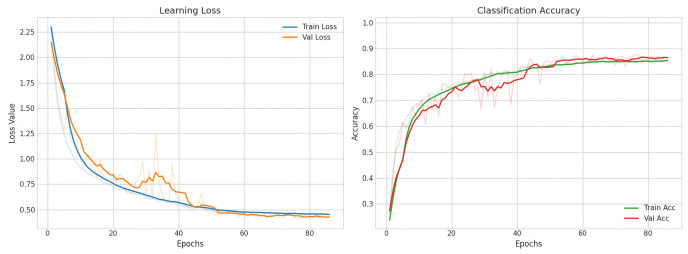
Training and validation results of the Multi-Scale CNN.

**Figure A4:**
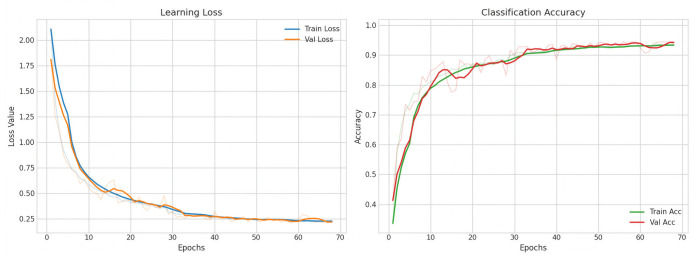
Training and validation results of the Multi-Channel CNN.

**Figure A5:**
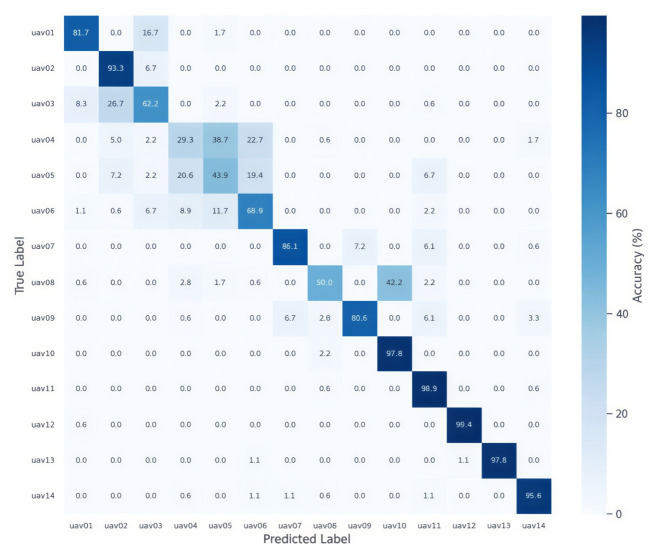
Confusion matrix of the Basic 1D CNN for UAV classification.

**Figure A6:**
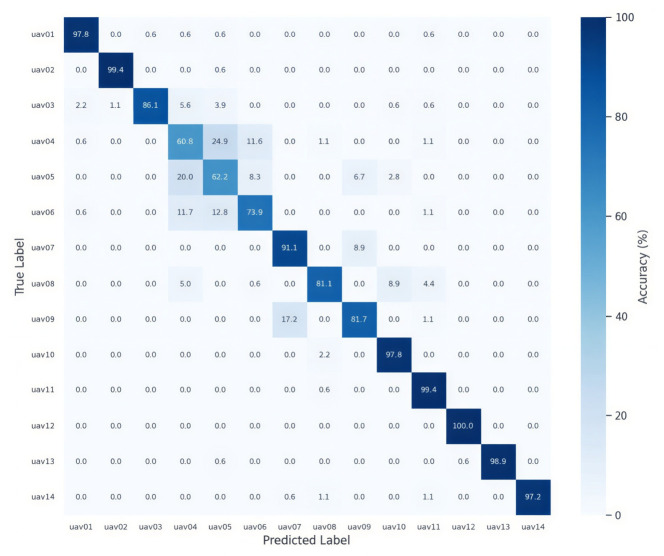
Confusion matrix of the Multi-scale CNN for UAV classification.

**Figure A7:**
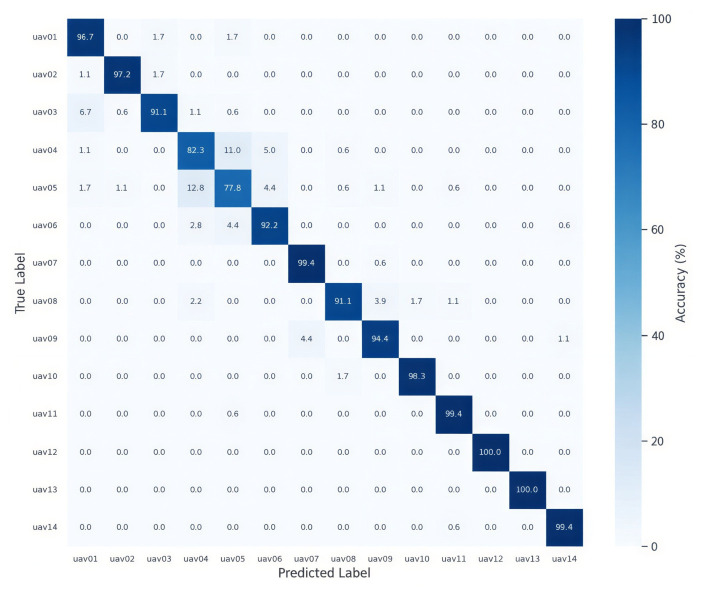
Confusion matrix of the Multi-Channel CNN for UAV classification.

**Figure A8:**
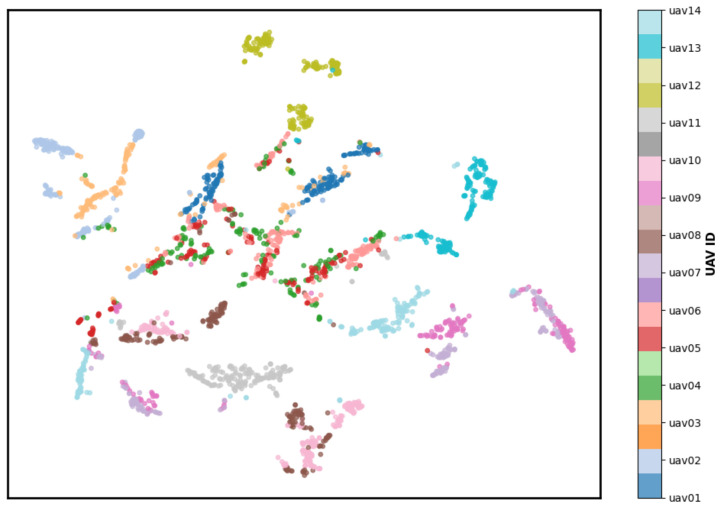
t-SNE visualization of feature embeddings for the Basic 1D CNN.

**Figure A9:**
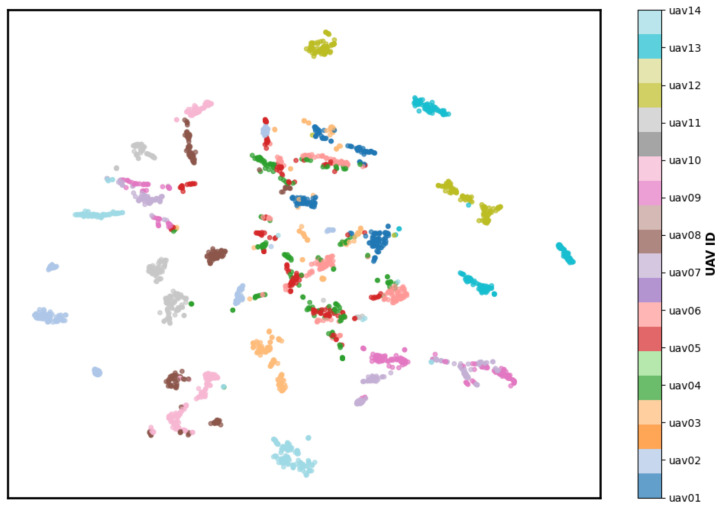
t-SNE visualization of feature embeddings for the Multi-Scale CNN.

**Figure A10:**
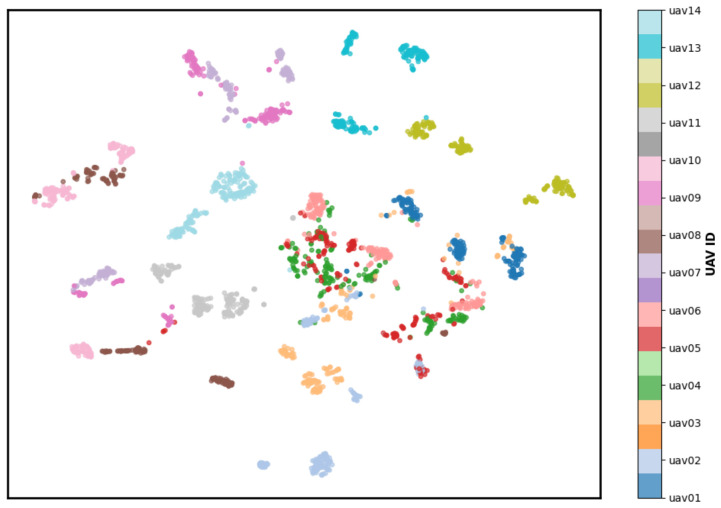
t-SNE visualization of feature embeddings for the Multi-Channel CNN.

## Data Availability

The full dataset used in this study is not publicly available at the time of publication because the dataset was constructed as part of an ongoing NIA data construction project and is subject to project-related disclosure regulations. The dataset is expected to be released through the AI Hub platform by the end of May 2026, upon completion of the required procedures. In the meantime, sample data and a README file have been made available for reference at the project GitHub repository: https://github.com/YoonMinJoo/LUMEN_Samples (accessed on 15 May 2026).

## Abbreviations

The following abbreviations are used in this manuscript:

UAV	Unmanned Aerial Vehicle
RF	Radio Frequency
RFFI	Radio Frequency Fingerprinting Identification
PSD	Power Spectral Density
CNN	Convolutional Neural Network
STFT	Short-Time Fourier Transform
SBC	Single-Board Computer
SDR	Software-Defined Radio
FHSS	Frequency Hopping Spread Spectrum
I/Q	In-phase and Quadrature
FFT	Fast Fourier Transform
SNR	Signal-to-Noise Ratio
NPU	Neural Processing Unit
ONNX	Open Neural Network Exchange
RKNN	Rockchip Neural Network
RAM	Random Access Memory
UHD	USRP Hardware Driver
FPS	Frames Per Second
DBI	Davies–Bouldin Index
CHI	Calinski–Harabasz Index
t-SNE	t-Distributed Stochastic Neighbor Embedding

## Appendix A. PSD Visualization of UAV Signals

To provide a comprehensive visual overview of the extracted spectral patterns, Figure A1 presents PSD examples for all 14 UAV models across five flight states. Each row corresponds to a UAV model (UAV01–UAV14), and each column corresponds to a flight state: arming, takeoff, landing, hovering, and moving. The figure illustrates both inter-class differences among UAV models and intra-class spectral variations across flight states.

As shown in Figure A1, several UAV models exhibit clearly distinguishable spectral characteristics, including different peak locations, bandwidth occupancy patterns, and sideband structures. In contrast, UAVs from similar device groups, particularly some DJI models, show partially similar spectral shapes, which may explain the localized confusion observed in the confusion matrix. In addition, limited flight-state-dependent variations are partially visible within the same UAV model, especially under moving conditions, where pitch, roll, and yaw maneuvers may induce additional temporal and spectral fluctuations. These observations provide qualitative support for the use of multi-channel PSD stacking and multi-scale feature extraction in the proposed LUMEN model.

## Appendix B. Detailed Model Architectures

In this part, we provide detailed architectural configurations of the four models used in the comparative experiments: Basic 1D CNN, Multi-Scale CNN, Multi-Channel CNN, and the proposed LUMEN. While the main text focuses on the design rationale of LUMEN, this appendix summarizes the layer-level configurations of all models to improve reproducibility and clarify their structural differences.

### Appendix B.1. Basic 1D CNN Architecture

Basic 1D CNN was implemented as a lightweight sequential one-dimensional convolutional baseline. It consists of two convolutional stages followed by global average pooling and a fully connected classifier.

**Table A1:** Detailed architecture of the Basic 1D CNN.

Block	Layer	Output Shape	Kernel/Stride/Pool	Parameters
Input	Input tensor	(1, 8192)	–	0
Block 1	Conv1d + BN + ReLU	(8, 8192)	k = 1, s = 1	32 + 16
	MaxPool1d	(8, 1024)	pool = 8, stride = 8	0
Block 2	Conv1d + BN + ReLU	(16, 1024)	k = 3, s = 1	400 + 32
	MaxPool1d	(16, 128)	pool = 8, stride = 8	0
Global pooling	AdaptiveAvgPool1d	(16, 1)	output = 1	0
Classifier	FC	(14)	16 → 14	238
Total				718

### Appendix B.2. Multi-Scale CNN Architecture

Multi-Scale CNN was designed to evaluate the effect of parallel kernel-size diversity. It employs three convolutional branches with different receptive fields, followed by feature concatenation and convolutional refinement.

**Table A2:** Detailed architecture of the Multi-Scale CNN.

Block	Layer	Output Shape	Kernel/Stride/Pool	Parameters
Input	Input tensor	(1, 8192)	–	0
Branch 1	Conv1d	(12, 8192)	k = 3, s = 1	48
Branch 2	Conv1d	(10, 8192)	k = 11, s = 1	120
Branch 3	Conv1d	(10, 8192)	k = 21, s = 1	220
Fusion	Concatenation	(32, 8192)	–	0
Refinement 1	BN + ReLU	(32, 8192)	–	64
	MaxPool1d	(32, 2048)	pool = 4, stride = 4	0
Refinement 2	Conv1d + BN + ReLU	(48, 2048)	k = 3, s = 1	4656 + 96
Global pooling	AdaptiveAvgPool1d	(48, 1)	output = 1	0
Classifier	FC	(14)	48 → 14	686
Total				5890

### Appendix B.3. Multi-Channel CNN Architecture

Multi-Channel CNN was constructed to examine the effect of using stacked adjacent PSD segments as multi-channel input in a one-dimensional CNN.

**Table A3:** Detailed architecture of the Multi-Channel CNN.

Block	Layer	Output Shape	Kernel/Stride/Pool	Parameters
Input	Input tensor	(3, 8192)	–	0
Block 1	Conv1d + BN + ReLU	(16, 8192)	k = 3, s = 1	160 + 32
	MaxPool1d	(16, 2048)	pool = 4, stride = 4	0
Block 2	Conv1d + BN + ReLU	(32, 2048)	k = 3, s = 1	1568 + 64
	MaxPool1d	(32, 512)	pool = 4, stride = 4	0
Block 3	Conv1d + BN + ReLU	(64, 512)	k = 3, s = 1	6208 + 128
Global pooling	AdaptiveAvgPool1d	(64, 1)	output = 1	0
Classifier	FC	(14)	64 → 14	910
Total				9070

### Appendix B.4. LUMEN Architecture

LUMEN combines multi-channel PSD input, multi-scale feature extraction, and hierarchical convolutional refinement in a lightweight architecture. Compared with the baseline models, it integrates both short-term spectral-context modeling and branch-wise receptive field diversity.

**Table A4:** Detailed architecture of the LUMEN model.

Block	Layer	Output Shape	Kernel/Stride/Pool	Parameters
Input	Input tensor	(3, 8192)	–	0
Branch 1	Conv1d	(8, 8192)	k = 3, s = 1	80
Branch 2	Conv1d	(8, 8192)	k = 11, s = 1	272
Branch 3	Conv1d	(8, 8192)	k = 21, s = 1	512
Fusion	Concatenation	(24, 8192)	–	0
Stage 1	BN + ReLU	(24, 8192)	–	48
	MaxPool1d	(24, 2048)	pool = 4, stride = 4	0
Stage 2	Conv1d + BN + ReLU	(48, 2048)	k = 3, s = 1	3504 + 96
	MaxPool1d	(48, 512)	pool = 4, stride = 4	0
Stage 3	Conv1d + BN + ReLU	(64, 512)	k = 3, s = 1	9280 + 128
Global pooling	AdaptiveAvgPool1d	(64, 1)	output = 1	0
Classifier	FC	(14)	64 → 14	910
Total				14,830

## Appendix C. Training and Validation Curves

Figure A2, Figure A3 and Figure A4 show the training and validation curves of the three baseline models: Basic 1D CNN, Multi-Scale CNN, and Multi-Channel CNN. All three models exhibited stable convergence without severe oscillation during training. The Basic 1D CNN reached a validation accuracy of approximately 79.5%, while the Multi-Scale CNN and Multi-Channel CNN achieved higher peak validation accuracies of 87.7% and 94.5%, respectively.

## Appendix D. Confusion Matrix Analysis

Figure A5, Figure A6 and Figure A7 show the confusion matrices of the three baseline models: Basic 1D CNN, Multi-Scale CNN, and Multi-Channel CNN. Overall, the baseline models showed different class-wise error patterns. The Basic 1D CNN exhibited relatively larger confusion among several DJI-manufactured UAV classes, particularly UAV04–UAV06. The Multi-Scale CNN reduced some of these errors, and the Multi-Channel CNN showed more stable class-wise performance across most classes.

## Appendix E. Feature Space Visualization via t-SNE

Figure A8, Figure A9 and Figure A10 present the t-SNE visualizations of the latent feature spaces for the three baseline models: Basic 1D CNN, Multi-Scale CNN, and Multi-Channel CNN. The Basic 1D CNN shows relatively loose clusters with noticeable overlap among several classes. The Multi-Scale CNN exhibits improved separation in some classes, although overlap remains in parts of the feature space. Among the baseline models, the Multi-Channel CNN shows the clearest cluster structure overall.
